# Neoantigens in Hematological Malignancies—Ultimate Targets for Immunotherapy?

**DOI:** 10.3389/fimmu.2019.03004

**Published:** 2019-12-20

**Authors:** Malte Roerden, Annika Nelde, Juliane S. Walz

**Affiliations:** ^1^Department of Hematology, Oncology, Rheumatology and Clinical Immunology, University Hospital Tübingen, Tübingen, Germany; ^2^Department of Immunology, Institute for Cell Biology, University of Tübingen, Tübingen, Germany; ^3^Clinical Collaboration Unit Translational Immunology, German Cancer Consortium (DKTK), University Hospital Tübingen, Tübingen, Germany

**Keywords:** neoantigens, hematological malignancies, mass spectrometry, immunopeptidomics, HLA antigens, NPM1 mutations

## Abstract

Neoantigens derive from non-synonymous somatic mutations in malignant cells. Recognition of neoantigens presented via human leukocyte antigen (HLA) molecules on the tumor cell surface by T cells holds promise to enable highly specific and effective anti-cancer immune responses and thus neoantigens provide an exceptionally attractive target for immunotherapy. While genome sequencing approaches already enable the reliable identification of somatic mutations in tumor samples, the identification of mutation-derived, naturally HLA-presented neoepitopes as targets for immunotherapy remains challenging, particularly in low mutational burden cancer entities, including hematological malignancies. Several approaches have been utilized to identify neoepitopes from primary tumor samples. Besides whole genome sequencing with subsequent *in silico* prediction of potential mutation-derived HLA ligands, mass spectrometry (MS) allows for the only unbiased identification of naturally presented mutation-derived HLA ligands. The feasibility of characterizing and targeting these novel antigens has recently been demonstrated in acute myeloid leukemia (AML). Several immunogenic, HLA-presented peptides derived from mutated Nucleophosmin 1 (NPM1) were identified, allowing for the generation of T-cell receptor-transduced NPM1^mut^-specific T cells with anti-leukemic activity in a xenograft mouse model. Neoantigen-specific T-cell responses have also been identified for peptides derived from mutated isocitrate dehydrogenase (IDH^mut^), and specific T-cell responses could be induced by IDH^mut^ peptide vaccination. In this review, we give a comprehensive overview on known neoantigens in hematological malignancies, present possible prediction and discovery tools and discuss their role as targets for immunotherapy approaches.

## Introduction

Recognition of tumor-associated antigens via human leukocyte antigen (HLA) molecules is pivotal for T cell-mediated tumor control and the induction of anti-tumor responses by immunotherapy (1). Neoantigens derive from non-synonymous somatic mutations and are of special interest, as they entail optimal tumor-specificity and lack central T-cell tolerance (2). These potentially highly immunogenic antigens are therefore considered prime targets for immunotherapy, particularly since neoantigens were described as targets of immune checkpoint inhibitor-induced anti-tumor T-cell responses (3, 4, 4–6). Recent advances in mass spectrometry (MS) (7, 8) and HLA antigen prediction algorithms (9–14) as well as the broad availability of whole genome sequencing (WGS) portrayed milestones in the field of cancer immunotherapy and hold promise to enable a robust and personalized identification of neoantigens in the future. The identification of spontaneous, neoantigen-specific T-cell responses in patients with long-lasting remissions suggests that neoantigen-specific targeting of tumor cells might enable durable anti-tumor responses (15–17). Long-lasting remissions could also be observed after personalized neoantigen-based peptide vaccination therapy in melanoma patients. Keeping the small sample size in these studies in consideration, these reports further indicate toward a therapeutic potential of neoantigens (18, 19). Following the success of checkpoint inhibitor therapy and the uncovering of the specificities of respective T-cell responses, a multitude of HLA-presented neoantigens have been identified in high mutational burden diseases such as melanoma (1, 3, 20). Hematological malignancies (HM) on the other hand are characterized by a low mutational burden and the role of neoantigens for immune-mediated tumor control and immunotherapeutic approaches in these entities remains to be elucidated. The immunogenicity of acute myeloid leukemia (AML) and other HM is demonstrated by the graft-vs.-leukemia/lymphoma effect and despite the typically low mutational burden, there has been steady progress in the identification of targetable neoantigens in these diseases in recent years. In this review we give an overview on known neoantigens in HM, their means of identification as well as the current state of efforts regarding the translation of these discoveries into the clinic.

## Identification of Patient-Specific Neoantigens

While genome sequencing approaches already allow for the reliable identification of patient-individual, tumor-specific mutations (1, 21), the subsequent identification of mutation-derived neoantigens remains challenging (1, 22). These novel targets can be present as mutated membrane proteins or, more frequently, as HLA-presented peptide fragments derived from intracellular proteins comprising the mutated sequence (1, 22). Frequently, identification of these HLA-presented neoepitopes is performed by *in silico* prediction of potential HLA binding motifs based on identified somatic mutations and subsequent confirmation of immunogenicity in *in vitro* T-cell assays by priming of naïve T cells or demonstration of pre-existing memory T-cell responses (1). However, as there is no direct correlation between genome, transcriptome, and immunopeptidome (23–25), this “reverse immunology approach” based on gene expression data and *in vitro* analyses can provide several “false positive” neoantigens lacking natural presentation on the tumor cell surface (18). This lack of correlation between gene expression and the immunopeptidome can be explained by the complex process of HLA ligand formation, which is furthermore frequently altered in tumor cells (26–29). Thus, only a very small fraction of predicted neoantigens is actually naturally processed and presented via HLA molecules on the tumor cell, calling for direct identification methods of HLA-presented neoepitopes to identify suitable targets for immunotherapy. This can be achieved by MS-based immunopeptidomics, which enables the only unbiased, in-depth analysis of the naturally presented HLA immunopeptidome (8, 30). Recent reports estimate, that only approximately one mutation-derived HLA-presented neoepitope arises from about 1,000 non-synonymous mutations (18, 22, 31–34). In HM, which are typically low mutational burden diseases with only a handful to a few hundred mutations (20), this implicates a low abundance or even absence of HLA-presented neoepitopes. Considering further that these can derive from passenger mutations, which are sensitive to immune escape mechanisms (1, 22) and are mainly patient-specific, the presence of broadly targetable neoantigens cannot be taken for granted in these diseases. Nevertheless, identification and successful targeting of recurrent and mainly driver mutation-derived neoantigens has recently been demonstrated in various HM (35–49) (Table 1), thereby expanding the prospects for immunotherapy in these entities.

**Table 1:** Overview of neoantigens in hematological malignancies.

**Hematological malignancy**	**Source protein of mutated neoantigen**	**Identification method**	**References**
AML	NPM1	MS, spontaneous CD8^+^ T-cell responses	(46, 50, 51)
	IDH 1	Spontaneous CD4^+^ T-cell responses	(44)
	IDH 2	MS	(51)
	FLT3	Spontaneous CD8^+^ T-cell responses	(38, 43)
	PML-RARα, DEK-CAN, ETV6–AML1	*In vitro* T-cell recognition	(52–54)
	*Splice variants:* NOTCH2, FLT3, CD44	Identification of transmembrane proteins	(35, 36)
CLL	ALMS1, C6ORF89, FNDC3B	Spontaneous CD8^+^ T-cell responses	(17)
CML	BCR-ABL	MS, spontaneous CD8^+^ T-cell responses	(41, 42, 55–59)
MCL	Ig heavy/light chain	MS, spontaneous CD4^+^ T-cell responses	(60)
MPN	JAK2	*In vitro* T-cell recognition	(48)
	CALR	*In silico* prediction, spontaneous CD4^+^ T-cell responses	(45, 47, 49, 61, 62)
	MPL	*In silico* prediction	(63)
Various	FBXW7	Spontaneous CD8^+^ T-cell responses	(45)
	MYD88	*In silico* prediction, *in vitro* T-cell recognition, spontaneous CD8^+^ T-cell responses	(40)

*AML, acute myeloid leukemia; ALMS1, Alstrom syndrome protein 1; CALR, calreticulin; CLL, chronic lymphocytic leukemia; CML, chronic myeloid leukemia; C6ORF89, chromosome 6 open reading frame 89; FBXW7, F-box/WD repeat-containing protein 7; FLT3, FMS like tyrosine kinase 3; FNDC3B, fibronectin type III domain containing 3B; IDH, isocitrate dehydrogenase; Ig, immunoglobulin; JAK2, janus like kinase 2; MCL, mantle cell lymphoma; MPL, myeloproliferative leukemia virus oncogene; MPN, myeloproliferative neoplasia; MS, mass spectrometry; MYD88, myeloid differentiation primary response protein 88*.

## Neoantigens in Acute Myeloid Leukemia

In AML, the mutational landscape is well-characterized (64) and several novel antigens derived from recurrent genetic alterations have been identified recently. Neoantigens derived from Nucleophosmin 1 mutations (NPM1^mut^), which occur in about 35% of AML patients (65), are arguably the most prominent targets in this regard. In most cases of NPM1^mut^ AML, a frameshift mutation in exon 12 leads to an altered c-terminal protein sequence, which can specifically be recognized by cytotoxic CD8^+^ T cells (66). It has been proposed, that the immunogenicity of NPM1^mut^ neoepitopes might add to the favorable prognosis of AML patients with NPM1 mutations (39). Several NPM1^mut^-derived HLA class I neoepitopes were identified by MS analysis in two recent studies (46, 50) and specific T-cell responses could be demonstrated in respective patients. Furthermore, isolation and transfer of a T-cell receptor (TCR) gene with an NPM1^mut^ neoepitope specificity was performed in one of these studies. TCR-transduced T cells subsequently showed anti-tumor efficacy in an AML xenograft mouse model, thereby emphasizing the potential of NPM1^mut^-specific T-cell-based immunotherapy approaches for the treatment of NPM1^mut^ AML (46). Missense mutations of isocitrate dehydrogenase (IDH) 1 or 2 can be detected in about 20% of AML patients, resulting in an altered, leukemogenesis promoting function of the enzymes (67). A study in glioma patients, where IDH mutations occur particularly frequent, identified an IDH1^mut^-derived HLA class II neoepitope and demonstrated its natural presentation and immunogenicity by detection of spontaneous CD4^+^ T-cell responses and mutation-specific antibodies in respective patients. CD4^+^ T-cell responses and mutation-specific antibody formation were subsequently induced by peptide vaccination in an HLA-humanized mouse model and led to IDH1^mut^-specific immune responses (44). Our own data support NPM1^mut^- and IDH^mut^-derived neoepitopes as promising targets in AML. Using our MS-based immunopeptidomics approach (24, 68–70), we were able to identify naturally presented HLA class I and II neoepitopes derived from mutated NPM1 and IDH2 in primary AML samples. Further analysis revealed multifunctional T-cell responses, and peptide-specific target cell killing was proven for one naturally presented NPM1^mut^ neoepitope (51). Additionally, AML-specific neoantigens can arise from internal tandem duplications (ITD) of the FMS like tyrosine kinase 3 (FLT3) gene, which occur in up to 30% of AML patients (71). While these duplications vary in length, the same protein domain is affected in the majority of cases (72). These mutations can yield immunogenic peptides, as described for a FLT3-ITD-derived HLA-A^*^01:01-restricted neoepitope showing specific T-cell responses *in vitro* as well as *ex vivo* (38, 43). A further source of HLA-presented neoepitopes are fusion proteins. In AML, *in vitro* T-cell recognition of fusion protein-derived HLA-presented peptides has been demonstrated for PML-RARα (52), DEK-CAN (53), and ETV6–AML1 (54). While these reports arouse interest in these potential targets, the clinical significance of these *in vitro* analyses remains to be elucidated as natural presentation and spontaneous immune responses against respective HLA-presented neoepitopes have not been demonstrated.

## Neoantigens in Chronic Myeloid Leukemia and Myeloproliferative Disorders

In chronic myeloid leukemia (CML), peptides encompassing the BCR-ABL fusion site in theory represent optimal targets for immunotherapy, as this fusion protein is essential for the malignant transformation, is present in virtually all CML cells and patients, and potentially provides HLA binding motifs. One major throwback however is the occurrence of several different fusion sites resulting in diverse mutation-derived peptides in distinct patients. The *t*(9;22) translocation mainly leads to the formation of an exon junction between exon 2 or 3 of *BCR* and exon 2 of *ABL* (*b2a2* and *b3a2*, respectively) (73). Several groups have described specific T-cell responses against HLA-presented peptides derived from b3a2 (41, 42, 55, 56). Furthermore, it has been demonstrated that BCR-ABL-specific T-cell responses can be induced with peptide vaccination in CML patients (57). One study reported the direct identification of a HLA-presented b3a2-derived neoepitope by MS on primary CML cells (58). However, in our recently performed extensive MS-based analysis of the primary CML immunopeptidome, we could not identify any naturally HLA-presented peptides encompassing BCR-ABL- or ABL-BCR-derived neoepitopes (68), keeping in mind that especially for MS-based immunopeptidomics, absence of evidence does not equal evidence of absence. Notably, neoantigens can also arise under therapy with tyrosine kinase inhibitors. In patients with imatinib-resistant CML, drug resistance-mediating mutations outside the BCR-ABL fusion site have been identified (59) and specific T-cell responses against neoepitopes derived from these mutations have been demonstrated and were linked with clinical response (59). Myeloproliferative disorders (MPN) are characterized by a homogenous mutational landscape with recurrent driver mutations, which in theory represent shared and therefore broadly applicable targets for immunotherapy. A single nucleotide mutation of the janus kinase 2 gene (JAK2 V416F) is the most frequent among MPN driver mutations, occurring in more than 90% of patients with Polycythemia vera (PV) and about 50% of patients with Essential thrombocytosis (ET) and Primary myelofibrosis (PMF), respectively (74). While experimental recognition of JAK2^mut^-derived neoepitopes has been demonstrated after *in vitro* priming of healthy donor T cells, thereby indicating the immunogenic potential, no spontaneous T-cell responses have been identified in JAK2^mut^ MPN patients (48). Direct identification of naturally presented JAK2^mut^ neoepitopes has not been reported so far. In MPN with Calreticulin mutations (CALR^mut^)—the most common driver mutation in JAK2 wildtype (JAK2^wt^) MPN, occurring in about 25% of ET and PMF patients (74)—a frameshift mutation leads to an altered c-terminus of the protein. Recent reports evaluated CALR^mut^-derived HLA-presented neoepitopes as targets for immunotherapy (45, 47, 49, 61, 62) as these peptides were predicted to bind HLA-A^*^03:01 and HLA-B^*^07:02. While natural presentation of these HLA class I neoepitopes could not be demonstrated upon MS analysis (45), spontaneous, primarily CD4^+^ T-cell responses against several CALR^mut^-derived neoepitopes could be identified in CALR^mut^ MPN patients (49). Further, CALR^mut^-dependent killing of autologous CALR^mut^ cells was demonstrated in an HLA-DR-restricted manner (47). Of note, while the observed CALR^mut^-specific T-cell responses in CALR^mut^ MPN patients were often weak (45, 49, 61), these could be restored by immune checkpoint blockade both *in vitro* and *in vivo* (61). These findings indicate that CALR^mut^ is naturally presented but respective T-cell responses are suppressed by immune checkpoint receptor signaling (61), illustrating the potential of combining distinct immunotherapeutic approaches for the treatment of HM. Taken together, the results revealed CALR^mut^-derived neoepitopes as shared MPN-specific neoantigens, prompting their further evaluation for therapeutic targeting (47, 49, 61, 62). Interestingly, CALR^mut^-specific memory T-cell responses were frequently detectable in healthy individuals in a subsequent study, suggesting a previous clearance of CALR^mut^ cells by immunosurveillant T cells and thereby further highlighting the immunogenic potential of CALR^mut^ neoantigens (62). Mutations in the myeloproliferative leukemia virus oncogene (MPL) are further recurrent driver mutations in JAK2^wt^ MPN (75). A recent study performing an *in silico* analysis based on whole transcriptome sequencing of MPN patients predicted several MPL^mut^ HLA class I binding neoepitopes (63). However, demonstration of natural presentation of MPL^mut^ neoepitopes by MS has not been performed thus far.

## Neoantigens in other Hematological Malignancies

FBXW7 is a tumor suppressor gene with mutations occurring in various HM, most frequently in T-ALL (76). Specific CD8^+^ T-cell responses against a recurrent FBXW7^mut^-derived neoepitope have been demonstrated, suggesting that this recurrent mutation might represent another neoantigen applicable for immunotherapy in HM (45). Mutation-derived neoantigens have also been identified in chronic lymphocytic leukemia (CLL). In a study, implementing a reverse immunology approach, immune responses were detected against HLA antigens derived from somatic mutations in ALMS1, C6ORF89, and FNDC3B (17). However, it should be kept in mind that these mutations are not considered driver mutations, thus theoretically making them suboptimal targets for immunotherapy. The identification of T-cell responses against these mutated neoantigens nevertheless demonstrated the applicability of neoantigen-specific targeting of CLL. Evaluation of neoantigens in mantle cell lymphoma patients applying a combined approach of whole exome sequencing and direct HLA ligand identification by MS revealed the presence of naturally presented HLA class II neoepitopes derived from the lymphoma immunoglobulin heavy- or light-chain variable regions (60). Spontaneous CD4^+^ T-cell responses could be identified against these neoepitopes and mediated tumor-specific killing of autologous lymphoma cells (60). MYD88 (L265P) is a recurrent driver mutation in Waldenstom's macroglobulinemia, CLL and other Non-Hodgkin lymphomas (77). We previously evaluated HLA class I neoepitopes derived from MYD88^mut^ as targets for immunotherapy in lymphoma patients (40). Based on *in silico* HLA motif prediction, further immunogenicity evaluation of possible MYD88^mut^-derived HLA class I neoepitopes was performed. *In vitro* priming of naïve T cells from MYD88^mut^ CLL patients and healthy donors was successful for several HLA-B:07- and HLA-B:15-restricted neoepitopes. While further analysis revealed that spontaneous MYD88^mut^-specific T-cell responses are infrequent in lymphoma patients, these *de novo* induced MYD88^mut^-specific T cells were multifunctional and elicited mutation-restricted cytotoxicity (40), highlighting the potential of MYD88^mut^ neoepitopes as targets for immunotherapy.

## Non-canonical Neoepitopes as Additional Tumor-Specific Targets

**Figure 1:**
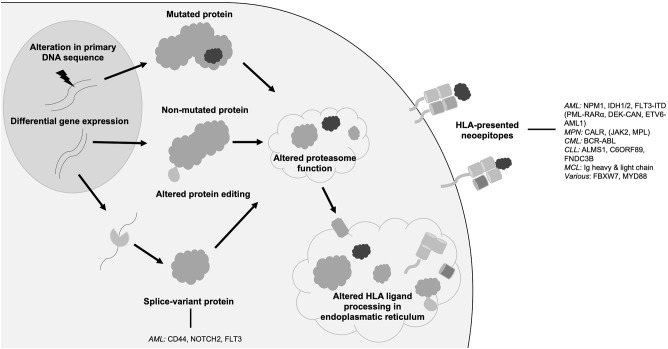
Origins of neoantigens. Schematic overview showing different origins of neoantigens with prominent examples in hematological malignancies. AML, acute myeloid leukemia; FLT3, FMS like tyrosine kinase 3; HLA, human leukocyte antigen; NPM1, nucleophosmin 1; IDH, isocitrate dehydrogenase; MPN, myeloproliferative neoplasia; CALR, calreticulin; JAK2 janus like kinase 2; CML, chronic myeloid leukemia; CLL, chronic lymphocytic leukemia; MCL, mantle cell lymphoma; Ig, immunoglobulin.

While the term *neoantigen* is mostly used referring to mutation-derived HLA-presented neoepitopes, the following section will discuss further sources of neoantigens that might also represent promising targets for immunotherapy. In distinction to mutations of the target's primary genome sequence, non-canonical neoantigens or cryptic peptides arise among others from tumor-specific alterations of the HLA antigen presentation machinery, DNA methylation, RNA editing or protein biosynthesis, proteasomal splicing, or non-canonical translation products (Figure 1) (29, 78–83). Further, tumor-specific splice variant proteins can result from splice-site creating mutations or mutations of spliceosome proteins and can lead to the formation of tumor-specific HLA-presented neoepitopes (84). Mutations directly affecting the spliceosome, such as SF3B1 or SRSF2 mutations, have been shown to occur in up to 20% of *de novo* AML (64) and 15% of PMF (63). In AML, frequently occurring splice variants have been identified for NOTCH2 (35), FLT3 (35), and CD44 (36), each leading to the occurrence of an altered cell surface protein. Specific targeting of a CD44 splice variant has been demonstrated by a CD44v6 CAR T cell in a mouse model (36), thereby highlighting the potential of targeting this class of neoantigens with immunotherapeutic approaches. Post-translational protein modifications, which are preserved in HLA-presented peptides, can portray another source of neoantigens as these tumor-specific alterations can lead to the formation of novel epitopes (29). In this regard, neoantigens resulting from tumor-specific phosphorylation as well as glycosylation have been identified in AML and immunogenicity of this class of neoantigens has been demonstrated (28, 37, 85, 86).

## Discussion

Neoantigen targeting holds promise to enable highly specific and durable anti-tumor immune responses (1, 22). Although HM are typically low mutational burden diseases (20), there has recently been remarkable progress in the uncovering of neoantigens in these entities. These discoveries were fueled by an immense progress in the field of WGS, steadily improved HLA motif prediction algorithms as well as technical advances in MS in recent years (1, 21, 31). While these advances already facilitate neoantigen identification from primary tumor samples, we are likely only seeing the beginning of personalized target evaluation. In this progress, a standard approach for target identification has yet to be defined (1, 22). As optimal target selection is a prerequisite for effective immunotherapy, we consider the direct identification of HLA-presented neoantigens by MS as the optimal approach. The direct identification of potential targets with MS harbors essential advantages when compared to the reverse immunology approach, which relies on neoantigen prediction, experimental HLA-binding and further immunogenicity confirmation in T-cell assays (1, 22). Here, “false targets” might be identified if HLA binding is demonstrated for predicted neoantigens *in vitro*, but these antigens are not naturally presented via HLA antigens *in vivo*. Furthermore, while the *ex vivo* identification of spontaneous T-cell responses against neoantigens can be regarded as evidence for natural HLA-presentation, potential targets without pre-existing responses might be missed. This is concerning as antigen-specific immunotherapy, including antibody strategies and peptide vaccines in particular aim to induce *de novo* anti-tumor responses. HM have been the first entities where immunotherapy—in form of allogenic stem cell transplantation (87)—has been performed and the immunogenicity of HM is long known (88, 89). While targeting of mutated membrane proteins by antibodies or CAR T cells has already been established for the treatment of HM, but is restricted to very few suitable surface antigens (90–94), HLA-presented neoantigens derived from intracellular proteins are of particular interest for immunotherapy. Hence, recent highly noted reports on the identification of neoantigens in HM have raised hopes that these novel targets might bring along new therapeutic options. In AML, NPM1^mut^- and IDH^mut^-derived neoantigens thus far represent the most promising targets, as these mutations occur frequently and successful as well as specific targeting has already been demonstrated in preclinical studies (44, 46, 51). Neoepitopes derived from fusion proteins are equally interesting targets, but natural presentation via HLA molecules has not been demonstrated so far (41, 42, 52–54, 56, 57, 95). While adoptive T-cell transfer and peptide vaccination approaches using non-mutated antigens are already under clinical evaluation in AML and other HM (96–98), the identification of these neoantigens will allow for an even more targeted approach in the future. However, it should be kept in mind that a personalized target selection remains challenging, as MS analysis is elaborate and not universally available. Furthermore, completely personalized immunotherapy approaches at the same time bring the difficulty of manufacturing an individualized product, e.g., a peptide vaccine, for each patient. A “warehouse” model, where a patient-specific selection of therapeutics targeting frequently occurring neoantigens can be made, might represent an elegant solution to this problem. This is particular true for malignancies with a well-characterized mutational landscape and a narrow spectrum of recurrent mutations, such as AML and MPN (20). At the same time and despite the recent progress in neoantigen identification in HM, the infrequency of individual mutations and HLA allotype restrictions still limit specific neoantigen targeting to a subset of patients. To overcome this issue, combined targeting of both mutated and non-mutated, tumor-exclusive antigens might be a suitable approach. Tumor-exclusive non-mutated neoepitopes can arise as a consequence of differential gene expression or tumor-specific alterations of RNA- and protein-processing, as described for splice variant proteins in AML (35, 36, 64) and have been shown to possess equally immunogenic properties as mutated neoepitopes (99). With mutations of spliceosome proteins, transcription factors and DNA methylation related proteins occurring frequently in AML and other HM (20, 64), additional non-canonical neoantigens are likely awaiting uncovering (35, 36, 84). Recent neoantigen discoveries have created novel and promising prospects for immunotherapy in HM. With currently ongoing endeavors, additional neoantigens might be uncovered and personalized target evaluation will be taken another step further. Considering the paucity of targetable mutated neoantigens in the individual patient due to HLA allotype restrictions and patient-individual mutations, combined approaches targeting both mutated and non-mutated tumor-exclusive antigens are likely warranted in patients with HM. Harnessing “the best of both worlds” might then enable immunotherapy to unfold its full potential in hematological malignancies.

## Author Contributions

MR wrote the first draft of the manuscript. AN and JW wrote further sections of the manuscript. All authors contributed to manuscript revision, read, and approved the final version.

### Conflict of Interest

The authors declare that the research was conducted in the absence of any commercial or financial relationships that could be construed as a potential conflict of interest.
